# Personalized approach for complex bilateral calcaneal osteomyelitis and defect reconstruction with bilateral abductor digiti minimi flaps

**DOI:** 10.1515/iss-2022-0010

**Published:** 2022-07-04

**Authors:** Oana Grigorescu, Adrian Dragu, Florian Bönke, Martin Schreiber, Stefan Rammelt

**Affiliations:** University Center for Orthopedics, Trauma and Plastic Surgery, Faculty of Medicine Carl Gustav Carus, Technische Universität Dresden, Dresden, Germany

**Keywords:** abductor digiti minimi muscle flap, calcaneal osteomyelitis, personalized approach

## Abstract

**Objectives:**

The best treatment for displaced, intra-articular fractures of the calcaneus remains controversial and it is generally agreed, that there is no single method that is suitable for all patients.

**Case presentation:**

Here we report a rare case of bilateral calcaneal osteomyelitis with fistula formation following open reduction and plate fixation via an extensile lateral approach that could be salvaged with an interdisciplinary approach including orthopedic and plastic surgeons. We are not aware of a similar case in the literature. Abductor digit minimi flaps is a well-established procedure in plastic and reconstructive surgery with a minimal functional defect and morbidity at the donor site. This treatment protocol resulted in minimal donor-site morbidity and good bone remodeling in the further course. We believe that it may be of use for complicated courses even with limited resources.

**Conclusions:**

Abductor digit minimi flaps is a well-established procedure in plastic and reconstructive surgery with a minimal functional defect and morbidity at the donor site.

## Introduction

The best treatment for displaced, intra-articular fractures of the calcaneus remains controversial and it is generally agreed, that there is no single method that is suitable for all patients. The treatment should be chosen individually based on the patient’s needs, the individual fracture pathoanatomy, the amount of soft tissue damage, associated injuries, and comorbidities. If operative treatment is chosen, reconstruction of the overall shape of the calcaneus and its joint surfaces are of the most important characteristic in order to obtain a good functional result [1], [2], [3], [4], [5].

The calcaneus is the greatest tarsal bone, and the sole posterior support of the foot providing a strong lever arm for the Achilles tendon and plantar fascia. Fractures of the calcaneus represent about 1–2% of all fractures and 60% of the tarsal fractures with 70% being-intra-articular [6]. The mechanism of injury can range from high-energy trauma (falls from a height, motor vehicle accidents) to low energy trauma (sports). The complex anatomy of the calcaneus and the vulnerable soft tissue cover including the unique heel pad imposes a challenge on the surgical team. Consequently, operative treatment is fraught with a considerable learning curve [3, 5, 7]. Following open reduction and internal fixation (ORIF) via an extensile lateral approach, wound healing problems and infections are reported in up to one third of patients [2, 5, 8]. The use of this flap is described in a calcaneal osteomyelitic case by Zgonis et al. in 2008 in a unilateral case.

Development of deep infection with osteomyelitis of the calcaneus represents a worst-case scenario and may lead to partial or total calcanectomy with serious permanent functional impairment of the affected patients [9, 10]. Therefore to avoid this kind of situations, a reconstructive concept should be chosen, one of them being the adbductor digiti minimi flap. The use of this flap has already been described in a calcaneal osteomyelitic case by Zgonis et al. in 2008. They used it in a unilateral case to cover a complicated wound with calcaneal osteomyelitis and wound dehiscence at the surgical incision.

## Case report

A 30 year-old man was referred to our trauma center with impaired wound healing and infection after multiple surgeries for bilateral intra-articular calcaneal fractures. Six months ago, he had jumped from a fire protection staircase at a height of about 2.5 m on a concrete surface while doing parkour sports. Initial fracture treatment consisted of bilateral open reduction and interlocking plate fixation via extensile lateral approaches. In the subsequent course, a deep infection developed on both sides necessitating multiple revisions with complete hardware removal, serial debridements and lavage until 4 months postoperatively. As part of this procedure, resorbable bone cement had been implanted. Because wound drainage and infection persisted, the patient had been offered bilateral partial calcanectomy. He therefore presented to our Foot & Ankle Center for a second opinion.

**Figure 1:**
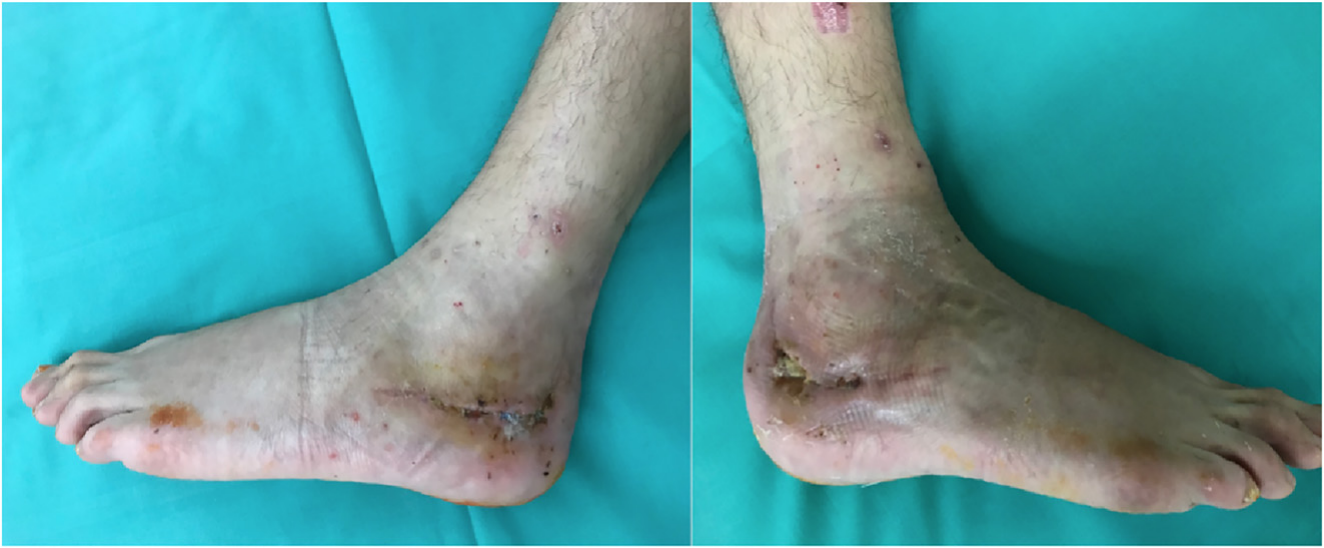
Clinical appearance of both feet at the time of initial presentation, 5 months after the injury, with fistula formation and continuous drainage from the angles of the extensile lateral approaches.

**Figure 2:**
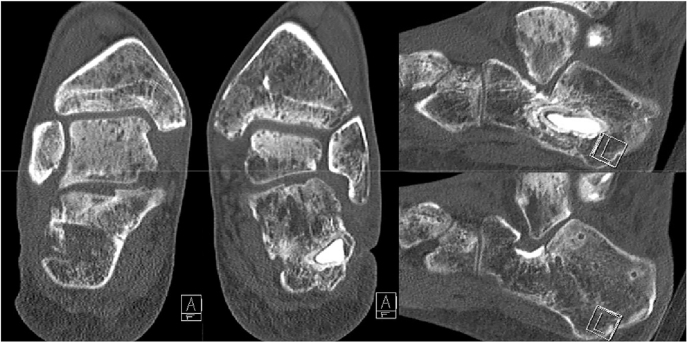
CT scans on admission showing healed bilateral calcaneal fractures with minimal residual step-off in the subtalar joint and a central osseous defect in both calcanei. On the right side, amorphous hydroxyapatite bone cement was in place that seemed encapsulated by a fibrous membrane.

For initial examination at our center, 6 months after the initial injury, the patient appeared in a wheelchair. The scars of the extensive lateral approaches over both heels displayed a fistula formation of almost 2 cm in the wound angles on both sides (Figure 1). With continuous effusion that was more pronounced on the right side than on the left side. Bone could be probed from the fistula on both sides. The peripheral blood circulation was intact. A hypesthesia was noted on the lateral aspect of both feet distal to the scar area and interpreted as sural nerve affection. The total range of motion of both feet displayed 30 degrees of plantarflexion and no dorsiflexion in the sagittal plane. Hindfoot motion was severely restricted with no eversion and inversion of 15° bilaterally. The subtalar joint appeared stiff on both sides. Lab results upon presentation included a leukocyte count of 6.87 GPt/L s and a C reactive protein (CRP) level of 30 mg/L. CT scans on admission revealed a heled calcaneal fracture with minimal residual step-off in the subtalar joint on both sides and a central osseous defect in both calcanei. On the right side, amorphous hydroxyapatite bone cement was in place that seemed encapsulated by a fibrous membrane (see Figure 2).

The patient was admitted to our hospital and after a detailed discussion of all possible treatment options.

An extended reconstructive therapy after infected ORIF of calcaneus fractures was established: we decided for a radical irrigation and debridement of all necrotic and infected bone and soft tissues, removal of the bone cement and insertion of a polymethylmethacrylate (PMMA) cement spacer with gentamycin (Palacos-G, Heraeus Inc., Hanau, Germany) and with vacuum assisted closure (3 M™ V.A.C., Germany) dressing to eradicate infection. There were in total seven debridements accomplished. Intraoperative swabs revealed multibacterial infection with *Staphylococcus epidermidis, Staphylococcus saprophyticus, Staphylococcus equorum, Enterococcus. avium* and *Enterococcus. faecalis*. The wound debridement’s were repeated until negative swabs were obtained.

**Figure 3:**
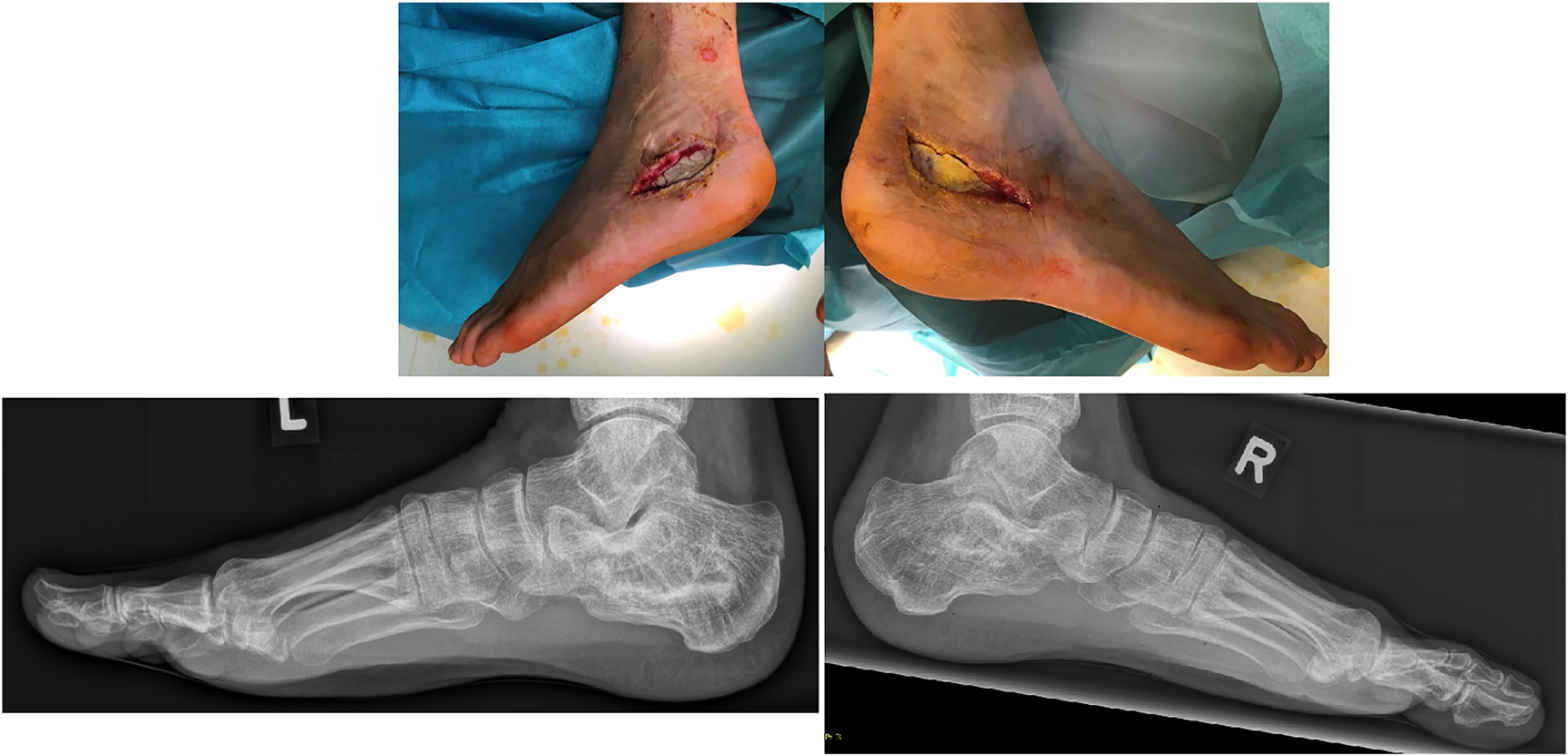
Clinical and radiological images following of the bone and soft tissue defects serial debridements, intermittent V.A.C. and implantation of a PMMA bone cement with gentamicin showing no residual signs of infection.

Additionally, an intravenous antibiotic first with Vancomycin for 10 days and then, matching the antibiogram, Amoxicillin was administered for 6 weeks intravenously. Following complete resection of all necrotic tissue, a bone defect remained measuring 20 × 19 × 14 mm on the right side and 57 × 25 × 14 mm on the left side (Figure 3). Furthermore, a full thickness soft tissue defect in the lateral hindfoot region 140 × 50 mm on the left and 150 × 70 mm right resulted, necessitating flap coverage (Figure 3).

Following negative cultures from multiple bone biopsies, reconstruction of the bone and soft tissue defect was performed 8 weeks following admission and first debridement. The osseous defects were filled with resorbable bone cement containing gentamicin sulfate (Cerament G, Bonesupport Inc., Lund, Sweden).

**Figure 4:**
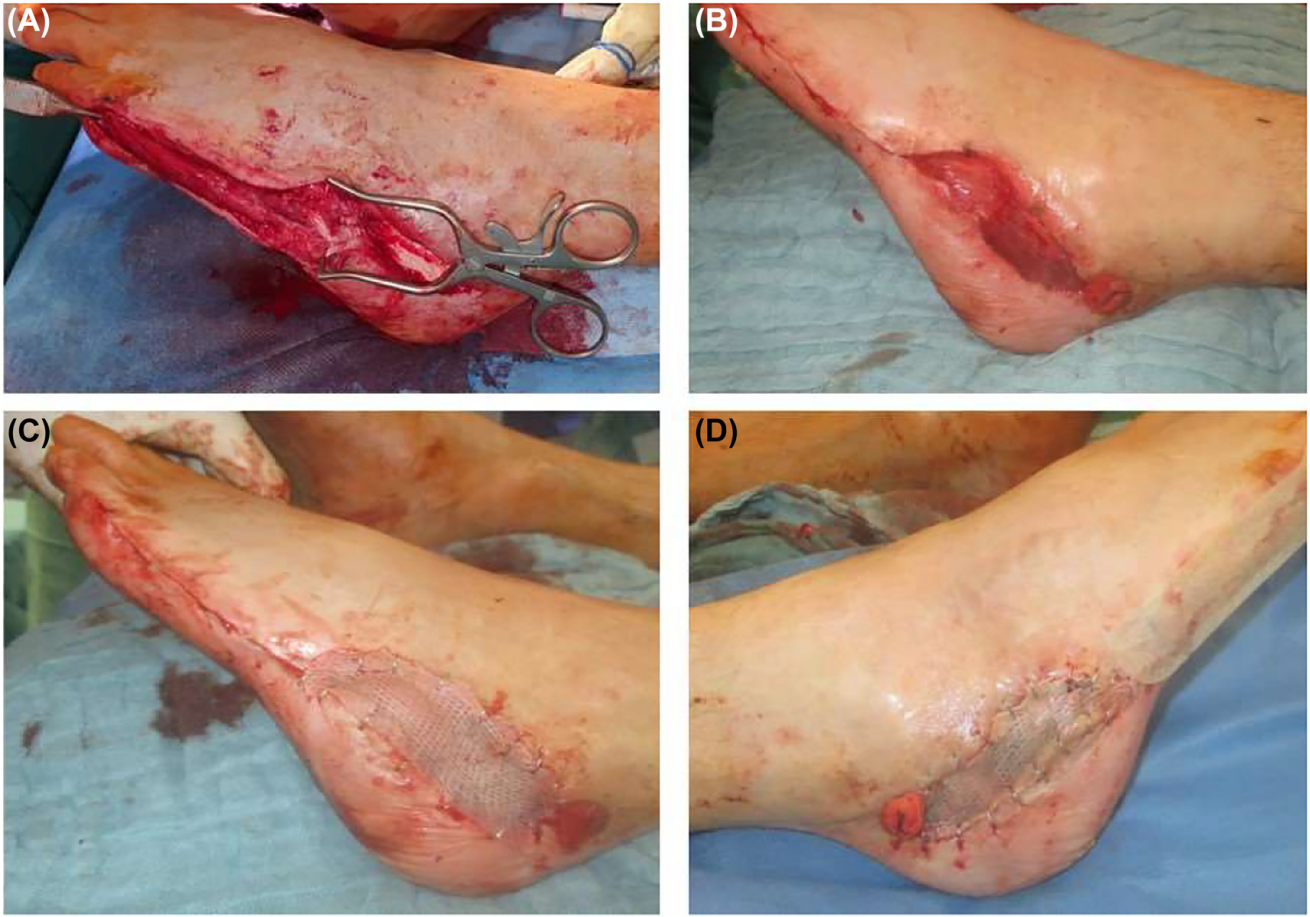
(A) The abductor digiti minimi flap is raised from its distal insertion at the left foot. (B) The muscle is pivoted at the level of the first perforator. (C) A skin graft from the thigh is used to cover the raised flap. (D) The defect on the right foot is covered with the same technique.

An abductor digiti minimi flap was raised on both feet to cover the soft tissue defects. The incision was carried out from the distal insertion of the abductor digitus quintus muscle at proximal phalanx of the fifth toe to the lateral aspect of the calcaneus at the transition from the glabrous to the hairy skin of the heel. The abductor digiti minimi muscle and tendon were identified and gently separated from the flexor digiti minimi muscle, technique also described in Wie Mardini, Flaps and Reconstructive Surgery (2nd Edition, pg. 818–823, 2009). The tendon was detached from the proximal phalanx of the fifth toe and held with a PDS suture for atraumatic handling. Dissection from the fifth metatarsal bone was continued proximally until the first perforators were reached. The wound edges were mobilized towards the lateral calcaneal wall. After mobilizing the muscle flap, it was pivoted around the perforators into the defect zone on the lateral calcaneus (Figure 4). Finally, the abductor digiti minimi muscle flap was covered with meshed split thickness skin graft from the thigh and sealed with a V. A. C. dressing that was removed after 4 days.

The patient was kept nonweight-bearing for 4 weeks and on partial weight-bearing for another 8 weeks in hindfoot-offloading Donjoy boots using two crutches. He returned to full weight-bearing after three months after prolonged clear drainage with particles of the bone substitute (“white washout”) for 10 weeks, the soft tissues healed on both sides.

**Figure 5:**
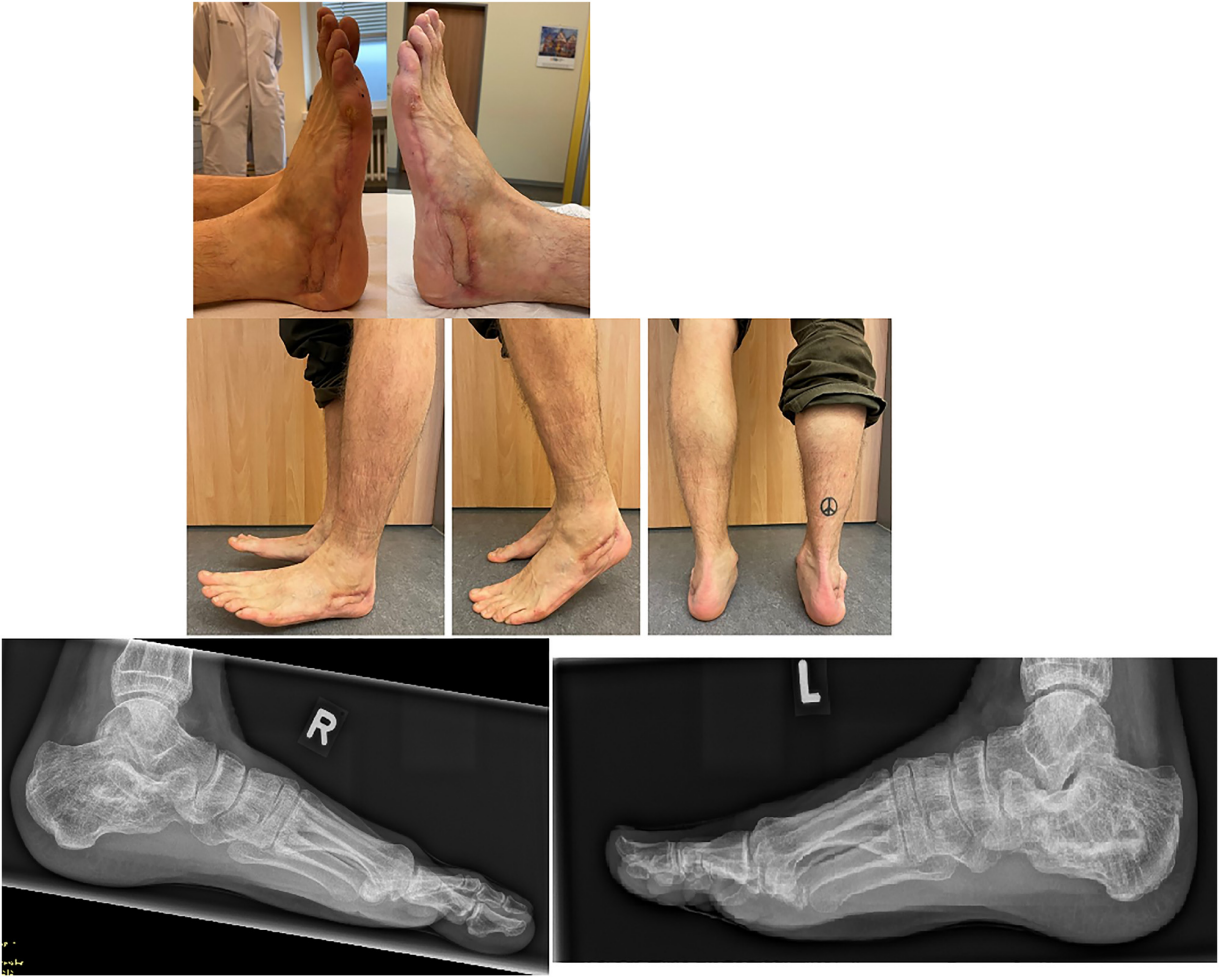
Radiological, clinical aspect and function at 1-year follow-up.

The patient was seen for a follow-up examination 9 months after defect filling and flap coverage. He was ambulating freely without crutches in sports shoes with insoles. He did not report pain when walking on even ground. The soft tissues had healed uneventfully without residual drainage on both feet (Figure 5). Range of motion was 45 degrees of dorsiflexion/plantarflexion in the sagittal plane and 20 degrees of eversion/inversion in the frontal plane. Except for the pre-existing hypesthesia, there were no neurovascular deficits. Standing radiographs including hindfoot alignment views revealed correct position of the hind foot and no signs of arthritis at the ankle, subtalar and calcaneocuboid joints. There was slight loss of heel height bilaterally. The bone cement was for the most part resorbed and replaced by cancellous bone.

## Discussion

Dislocated intra-articular calcaneus fracture represent a challenge for surgeons and wound healing problems are not unfrequently following the use of an extensile lateral approach. These are associated with increased morbidity and long periods of inactivity. Bilateral calcaneal fractures, making up 8% of all calcaneal fractures [8], are particularly demanding with respect to management [5] and bilateral deep infection with osteomyelitis represents a worst-case scenario for the patient. Soft tissue defects around the calcaneus are difficult to treat because of the vulnerable but unique soft tissue cover of the heel. The treatment of combined bone and soft tissue defects is extremely challenging. Pedicled or free tissue transfer may be required to avoid calcanectomy which has a profound impact on global foot function [9, 10]. Due to the irregular calcaneal anatomic shape, complex biomechanics of adjacent joints, and delicate soft tissue cover, in order to create an individual treatment concept, interdisciplinary management by orthopedic and plastic surgeons is indispensable in these cases [10].

The complex care of bone and soft tissue defects reconstruction with abductor digiti minimi flap after an open reduction and internal fixation of a severely comminuted calcaneal fracture was also described by Zgonis et al. in 2008.

Abductor digiti minimi flaps is a well-established procedure in plastic and reconstructive surgery with a minimal functional defect and morbidity at the donor site [9]. Nevertheless, it represents a good technique to cover lateral heel defects resulting from debridement after chronic osteomyelitis of the calcaneus with fistula formation. The bilayer cover of muscle and skin graft offers a sufficient stability and pliability of the vulnerable lateral aspect of the heel [6]. This kind of flap can also be used for the treatment of nonhealing ulcers in patients with diabetes after carefully analysis of the anatomic location, dimension and the comprehension of the other tissue involvement [7]. It is a less demanding procedure than a free microvascular flap with less donor site morbidity. On the other hand, it provides a more stable and pliable soft tissue cover than split thickness skin grafting alone.

Another treatment options for salvage at the critical situation of bilateral and symmetrical appearance but also for the filling large of bone defects following calcaneal osteomyelitis and debridement. These include calcium phosphate-based bone cements or hydroxyapatite-based ceramics with and without antibiotics, autologous and allogeneic bone grafting, muscle flaps, and composite vascularized bone and soft tissue transfer for combined lesions [9].

For the present case, temporary implantation of a PMMA bone cement with antibiotics helped with resolution of the infection and induction of a periost-like membrane to promote bone healing (Masquelet technique). For definitive defect filling, bone cement with continuous release of antibiotics was chosen following a multigerm infection. Using a synthetic, resorbable bone substitute avoids donor site morbidity that is invariably accompanying either free or pedicled autologous bone grafts.

In summary, we report a case of bilateral posttraumatic calcaneal osteomyelitis with fistula formation and full thickness skin defects that was successfully salvaged with serial debridements, temporary filling with PMMA cement and vacuum assisted wound closure, followed by defect filling with resorbable bone substitute with continuous release of antibiotics and personalized reconstruction with pedicled abductor digiti minimi flaps covered with split thickness skin grafts. We are not aware of any similar case in the literature. This treatment protocol resulted in minimal donor site morbidity and good bone remodeling in the further course. We believe that it may be of use for complicated courses even with limited resources.

## Supplementary Material

Supplementary Material
