# Supplementary material for: Personalized approach for complex bilateral calcaneal osteomyelitis and defect reconstruction with bilateral abductor digiti minimi flaps
Source: Innov Surg Sci. 2022 Jul 4;7(2):65–70. doi: 10.1515/iss-2022-0010 (PMC9574652; doi:10.1515/iss-2022-0010)
Supplement: Supplementary file 1 — Supplementary Material [file j_iss-2022-0010_suppl.pdf]

Reviewer Assessment

O. Grigorescu et al.: Personalized approach for complex bilateral calcaneal osteomyelitis

Reviewers' Comments to Original Submission

Reviewer 1: Geerling, Jens

Date received: 12-May-2022
Reviewer recommendation: **Return to author for minor modifications**
Reviewer overall scoring: **High**

Assessment Form scores: 5 = High/Yes; 3 = Medium/Adequate; 1 = Low

Is the subject area appropriate for the journal	5				
Does the title clearly reflect the paper's content?			3		
Does the abstract clearly reflect the paper's content					
Do the keywords clearly reflect the paper's content?			3		
Does the introduction present the problem clearly?			3		
Are the results/ conclusions justified?		4			
How comprehensive and up-to-date is the subject matter presented?		4			
How adequate is the data presentation?		4			
Are units and terminology used correctly?		4			
Is the number of cases adequate?		4			
Are the experimental methods/ clinical studies adequate?					
Is the length appropriate in relation to the content?		4			
Does the reader get new insights from the article?			3		
Please rate the practical significance.			3		
Please rate the accuracy of methods.					
Please rate the statistical evaluation and quality control.					
Please rate the appropriateness of the figures and tables.			3		
Please rate the appropriateness of the references.			3		
Please evaluate the writing style and use of language.		4			
Please judge the overall scientific quality of the manuscript.		4			
Are the methods used worthy of reproduction in greater deal?	Yes				
Would you be willing to review a revision of this manuscript?	Yes				

Comments to author: The article describe the use of an abductor digiti minimi flap for an bilateral osteomyelitis of the calcaneus. Due to a quick research on pubmed it is the first describtion of the use in a bilateral case. However, the use of this flap is described in a calcaneal osteomyelitic case by Zgonis et al in 2008 in a unilateral case. I think this should be mentioned within the introduction.

Furthermore, to get a direct idea of the article, the coverage should be mentioned within the title.
Additionally the rest will be mentioned line by line.
Case Report,
page 7, line 21/22: The company of the V.A.C. should be mentioned.
page 7, line 24: how many debridements were neccessary?
page 7, line 25: for how many days vancomycin was administered,
page 7, line 26: the amoxicillin was administered for 6 weeks intravenous?
page 7, line 28: Fig 3 only shows a clinical image, no x-ray; this should be shown additionally.
page 7, line 42: is it possible to support literature for the procedure? e.g. as a quote
page 7, line 49: please give details of the boots (e.g. Walker, Donjoy with....)
page 8, line: The radiographs are not shown in any illustration.

The reviewer think, that the radiographs are as interesting as the clinical illustrations. Maybe it is possible to create the illustration of the clinical result in combination with the radiographic result to keep 5 images.

Within the discussion one classical paper for calcaneal fractures should be sufficient. so the paper of zgonis can be mentioned..

Reviewer 2: anonymous

Date received: 18-May-2022
Reviewer recommendation: **Accept in present form**
Reviewer overall scoring: **High**

Assessment Form scores: 5 = High/Yes; 3 = Medium/Adequate; 1 = Low

Is the subject area appropriate for the journal		4			
Does the title clearly reflect the paper's content?	5				
Does the abstract clearly reflect the paper's content	5				
Do the keywords clearly reflect the paper's content?	5				
Does the introduction present the problem clearly?	5				
Are the results/ conclusions justified?		4			
How comprehensive and up-to-date is the subject matter presented?		4			
How adequate is the data presentation?		4			
Are units and terminology used correctly?	5				
Is the number of cases adequate?			3		
Are the experimental methods/ clinical studies adequate?			3		
Is the length appropriate in relation to the content?	5				
Does the reader get new insights from the article?		4			
Please rate the practical significance.	5				
Please rate the accuracy of methods.	5				
Please rate the statistical evaluation and quality control.			3		
Please rate the appropriateness of the figures and tables.	5				
Please rate the appropriateness of the references.	5				
Please evaluate the writing style and use of language.		4			
Please judge the overall scientific quality of the manuscript.		4			

Are the methods used worthy of reproduction in greater deal?	Yes
Would you be willing to review a revision of this manuscript?	Yes

Comments to author: Precise description of an extended reconstructive therapy after infected ORIF of calcaneus fractures. Novel the bilateral and symetical appearance at the compilation. Good review of treatment options for salvage at the critical situation.

Authors' Response to Reviewer Comments

Date received: 21-May-2022

Response to reviewer 1

Comments to the Author:

- I added in the introduction, that this flap was already described in 2008:

Therefor to avoid this kind of situations, a reconstructive concept should be chosen, one of them being the adbductor digiti minimi flap. The use of this flap has already been described in a calcaneal osteomyelitic case by Zgonis et al in 2008. They used it in a unilateral case to cover a complicated wound with calcaneal osteomyelitis and wound dehisence at the surgical incision.

- page 7, line 21/22: The company of the V.A.C. should be mentioned

(3M™ V.A.C., Germany)

- page 7, line 24: how many debridements were neccessary?

There were in total 7 debridements accomplished

- page 7, line 25: for how many days vancomycin was administered?

Vancomycin was administered for 10 days

- page 7, line 26: the amoxicillin was administered for 6 weeks intravenous?

Amoxicillin was administered for 6 weeks intravenously

- page 7, line 28: Fig 3 only shows a clinical image, no x-ray; this should be shown additionally.

X-Rays added

- page 7, line 42: is it possible to support literature for the procedure? e.g. as a quote

technique also described in Wie Mardini, Flaps and Reconstructive Surgery (2nd Edition, pg. 818-823, 2009)

- page 7, line 49: please give details of the boots (e.g. Walker, Donjoy with….)

hindfoot-offloading Donjoy boots

- page 8, line: The radiographs are not shown in any illustration.

X-Rays added

- Within the discussion one classical paper for calcaneal fractures should be sufficient. so the paper of zgonis can be mentioned.

The complex care of bone and soft-tissue defects reconstruction with abductor digiti minimi flap after an open reduction and internal fixation of a severly comminuted calcaneal fracture was also described by Zgonis et al in 2008..

Response to reviewer 2

Precise description of an extended reconstructive therapy after infected ORIF of calcaneus fractures.

An extended reconstructive therapy after infected ORIF of calcaneus fractures was established: we decided for a radical irrigation and debridement of all necrotic and infected bone and soft tissues, removal of the bone cement and insertion of a polymethylmethacrylate (PMMA) cement spacer with gentamycin (Palacos-G, Heraeus Inc., Hanau, Germany) and with vacuum assisted closure (3M™ V.A.C., Germany) dressing to eradicate infection. There were in total 7 debridements accomplished. Intraoperative swabs revealed multibacterial infection with Staphylococcus epidermidis, Staphylococcus saprophyticus, Staphylococcus equorum, Enterococcus. avium and Enterococcus. faecalis. The wound debridement's were repeated until negative swabs were obtained.

Additionally, an intravenous antibiotic first with Vancomycin for ten days and then, matching the antibiogram, Amoxicillin was administered for 6 weeks intravenously. Following complete resection of all necrotic tissue, a bone defect remained measuring 20 x 19 x 14 mm on the right side and 57 x 25 x 14 mm on the left side (Fig. 3). Furthermore, a full thickness soft tissue defect in the lateral hindfoot region 140 x 50 mm on the left and 150 x 70 mm right resulted, necessitating flap coverage (Fig. 3).

Novel the bilateral and symetical appearance at the compilation. Good review of treatment options for salvage at the critical situation.

Another treatment options for salvage at the critical situation of bilateral and symetical appearance but also for the filling large of bone defects following calcaneal osteomyelitis and debridement. These include calcium phosphate-based bone cements or hydroxyapatite-based ceramics with and without antibiotics, autologous and allogenic bone grafting, muscle flaps, and composite vascularized bone and soft tissue transfer for combined lesions [9].

Comments by the Editor-in-Chief to Revised Submission

All reviewer comments were addressed adequately and the manuscript should be published in its present stage.
